# ﻿A new troglomorphic species of *Larca* (Pseudoscorpiones, Larcidae) from Colorado

**DOI:** 10.3897/zookeys.1198.120353

**Published:** 2024-04-25

**Authors:** Mark S. Harvey, David B. Steinmann

**Affiliations:** 1 Collections & Research, Western Australian Museum, 49 Kew Street, Welshpool, Western Australia 6106, Australia Western Australian Museum Welshpool Australia; 2 School of Biological Sciences, University of Western Australia, Crawley, Western Australia 6009, Australia University of Western Australia Crawley Australia; 3 Department of Zoology, Denver Museum of Nature & Science, 2001 Colorado Boulevard, Denver, Colorado 80205, USA Denver Museum of Nature & Science Denver United States of America

**Keywords:** morphology, Nearctic, new species, pseudoscorpion, taxonomy, troglomorphic

## Abstract

A new species of *Larca* is described from dry habitats in a cave in central Colorado. Like other cave-dwelling species of *Larca*, the new species *Larcaboulderica***sp. nov.**, shows relatively modest morphological adaptations, such as pale colouration and slightly elongated appendages, compared with their epigean counterparts. This species is the sixth cave-dwelling species of *Larca* described from North America and, like other cave-dwelling *Larca* in North America and Europe, tends to be distributed in more southerly regions.

## ﻿Introduction

The pseudoscorpion family Larcidae consists of only 15 species found throughout Europe and North America. Although the family was traditionally divided into two genera with *Larca* Chamberlin, 1930 distinguished from *Archeolarca* Hoff & Clawson, 1952 only in the number of trichobothria on the movable chelal finger (*Larca* with 2 or 3 trichobothria and *Archeolarca* with 4 trichobothria), these genera were regarded as synonyms by Harvey and Wynne (2014). The European fauna consists of *L.bosselaersi* Henderickx & Vets, 2002, *L.fortunata* Zaragoza, 2005, *L.hispanica* Beier, 1939, *L.italica* Gardini, 1983, *L.lata* (Hansen, 1884) and *L.lucentina* Zaragoza, 2005, and the North American fauna consists of *L.aalbui* (Muchmore, 1984), *L.cavicola* (Muchmore, 1981), *L.chamberlini* Benedict & Malcolm, 1978, *L.granulata* (Banks, 1891), *L.guadalupensis* (Muchmore, 1981), *L.laceyi* Muchmore, 1981, *L.notha* Hoff, 1961, *L.rotunda* (Hoff & Clawson, 1952) and *L.welbourni* (Muchmore, 1981).

*Larca* was originally treated as a member of the family Garypidae by Chamberlin (1930) until both it and *Archeolarca* Hoff & Clawson, 1952 were transferred to their own family by Harvey (1992). A recent phylogenomic study found that Larcidae are sister to Garypinidae, and that they belong to their own superfamily, Garypinoidea, which in turn is sister to a larger clade of Cheiridioidea + Sternophoroidea + Cheliferoidea (Benavides et al. 2019). A multi-gene analysis of larcids and garypinids found that *Larca* nested within Garypinidae (Harvey 2023).

Among some recently collected cave-dwelling pseudoscorpions from Colorado were specimens of Larcidae that differed in several ways from other species of *Larca*. That species is described here.

## ﻿Materials and methods

The specimens examined for this study are lodged in the Denver Museum of Nature & Science, Colorado (DMNS) and the Western Australian Museum, Perth (WAM). They were studied using temporary slide mounts prepared by immersion of the specimens in lactic acid at room temperature for several hours, and mounting them on microscope slides with a 10 mm coverslip supported by small sections of 0.25 mm diameter nylon fishing line. After the study, the specimens were rinsed in water and returned to 75% ethanol with the dissected portions placed in 12 × 3 mm glass genitalia microvials (BioQuip Products, Inc.). The specimens were examined with a Leica MZ16 A dissecting microscope and an Olympus BH2 compound microscope, and illustrated with the aid of a drawing tube attached to the compound microscope. Measurements were taken at the highest possible magnification using an ocular graticule.

Terminology and mensuration mostly follow Chamberlin (1931), with the exception of the nomenclature of the pedipalps, legs and some minor modifications to the terminology of the trichobothria (Harvey 1992), chelicera (Judson 2007) and faces of the appendages (Harvey et al. 2012).

### ﻿Ecology

**Figure 1.**
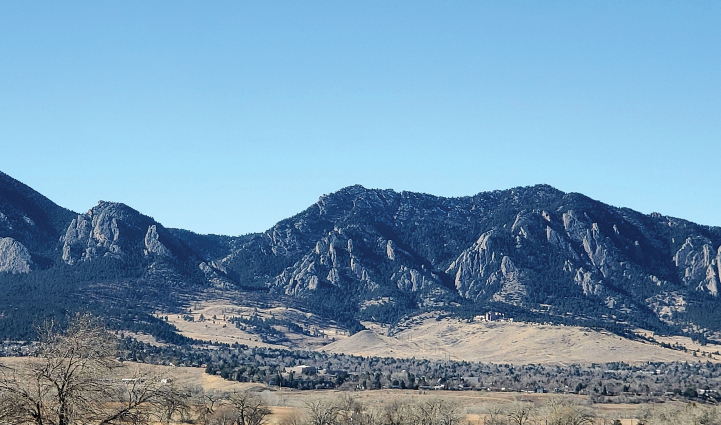
The foothills of Boulder where Mallory Cave is located.

The type locality, Mallory Cave, is at the eastern edge of the Rocky Mountains in the foothills of Boulder County, Colorado (Fig. 1). The cave is on Dinosaur Mountain to the west of City of Boulder on City of Boulder Open Space and Mountain Parks land. Mallory Cave formed in the Fountain Formation which is a sandstone conglomerate that was deposited approximately 280 Ma during the Pennsylvanian Period (Evanoff and Hirschfeld 2016).

Mallory Cave consists of one large room 25 m wide by 7 m deep with a single walk-in entrance that faces east. The temperature inside is 55 °F (13 °C). The cave is gated to protect a maternity colony of Townsend’s big-eared bats (*Corynorhinustownsendii* Cooper, 1837). There were less than 10 bats roosting in Mallory Cave in 2008 and 2009. Over 60 Townsend’s big-eared bats were documented roosting in the cave in 2023 (B. Stoner, personal communication, 20 March 2024). The cave was gated in 2011 which limited human disturbances to the bats and helped the bat colony increase in size. The *Larca* specimens were collected in the fall of 2008 and 2009 after the bats left the cave for the winter and no guano was observed.

The *Larca* specimens were found among the remnants of packrat nests and under rocks in arid and dusty areas in the dark zone of the cave. They were clustered in groups of 4–10 individuals. Mallory Cave is dry in the southern section where the *Larca* specimens were collected. The western and northern sections of the cave have ceiling drips, wet seeping walls, and a moist floor further inside.

Packrats that use Mallory Cave are bushy-tailed woodrats (*Neotomacinerea*) which is the only packrat species known from Boulder County (Armstrong et al. 2011). The packrat nest remnants were 3–5 cm deep and 30 cm in diameter consisting of scattered debris from abandoned nests. The nest remnants were visually searched for invertebrates. They contained leaves, sticks, pine cones, grasses and fresh packrat scat. Tweezers were used to move the nesting materials around while looking for invertebrates. There are no large packrat middens in the cave. No packrats were observed, and no lice or fleas were seen in the packrat nest remnants.

There are a few smaller caves located near Mallory Cave that were not searched for invertebrates, including Harmon Cave and Bear Cave, which could harbour populations of *L.boulderica* sp. nov. Deep cracks in the local rock formations, plus nearby boulder talus fields and packrat nests, may also provide habitat for *L.boulderica*.

Other invertebrates living in Mallory Cave include springtails, harvestmen, spiders, flies, beetles, centipedes and mites. No fleas or lice, which can be associated with rodents, were observed in the cave. Mice (*Peromyscus* sp.) may enter Mallory Cave, though no evidence of mice, including mouse scat or nests, was seen. Guano from Townsend’s big-eared bats provide organic nutrient input for the invertebrates inhabiting the cave.

### ﻿Biogeography

**Figures 2, 3.**
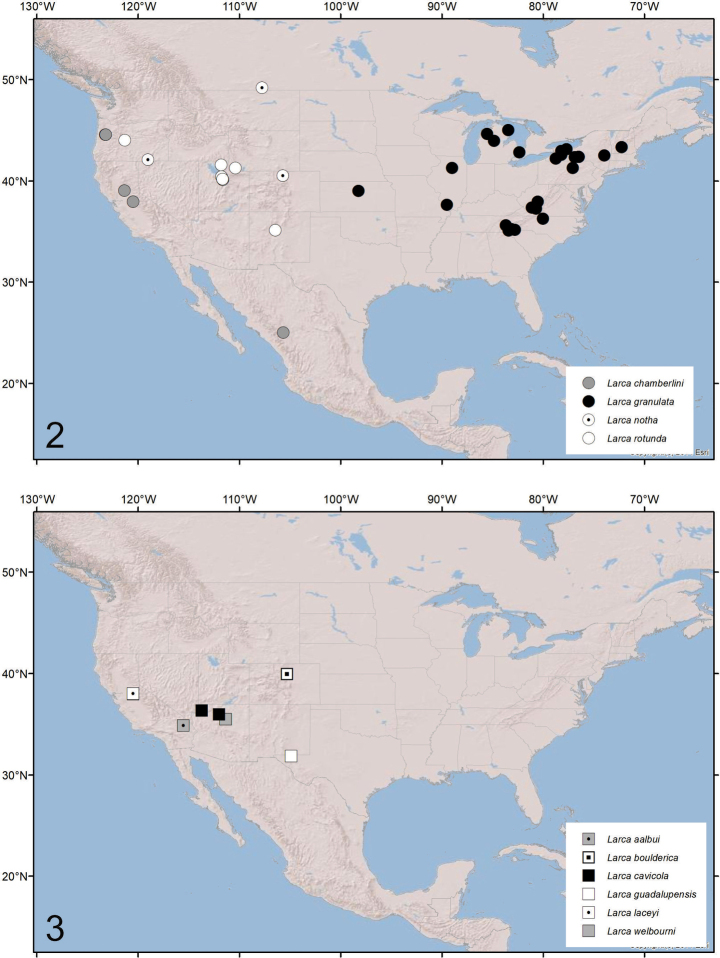
Distribution of *Larca* in North America: **2** epigean species **3** cave-dwelling species.

With the description of *Larcaboulderica*, the North American larcid fauna now comprises 10 species. Four are rather widely distributed in epigean habitats: *L.granulata* occurs across a wide variety of habitats from the mid-west to New Hampshire (Muchmore 1981; World Pseudoscorpiones Catalog 2023); *L.rotunda* from New Mexico, Oregon, Utah and Wyoming (e.g., Hoff and Clawson 1952; Muchmore 1981), *L.notha* in Colorado, Oregon and southern Canada (e.g., Hoff 1961; Benedict and Malcolm 1978; Muchmore 1981), and *L.chamberlini* in Oregon, California and Mexico (e.g., Benedict and Malcolm 1978; Villegas-Guzmán and Pérez 2005) (Fig. 2). The other six species appear to be obligate cave-dwelling forms with morphological modifications that are indicative of troglomorphic traits. The pedipalps and legs are slightly longer and thinner than their epigean counterparts, they are paler, and the eyes are reduced in size. They appear to represent short-range endemic species with highly restricted distributions: *L.aalbui* from Mitchell Caverns, California (Muchmore 1984), *L.cavicola* from Grand Canyon National Park and Parashant National Monument, Arizona (Muchmore 1981; Harvey and Wynne 2014), *L.guadalupensis* from Guadalupe Mountains National Park, Texas (Muchmore 1981), *L.laceyi* from Music Hall Cave, California (Muchmore 1981) and *L.welbourni* from Wupatki National Monument, Arizona (Muchmore 1981) (Fig. 3). There is a single record of *L.chamberlini* from a cave in Calaveras County, California but Muchmore (1981) surmised that it was only accidentally found in the cave.

Whereas the epigean species generally occur across a wide range of habitats in North America (Fig. 2), the subterranean species occur in more southerly regions in Arizona (*L.cavicola* and *L.welbourni*), California (*L.aalbui* and *L.laceyi*), Colorado (*L.boulderica*) and Texas (*L.guadalupensis*) (Fig. 3).

**Figures 4, 5.**
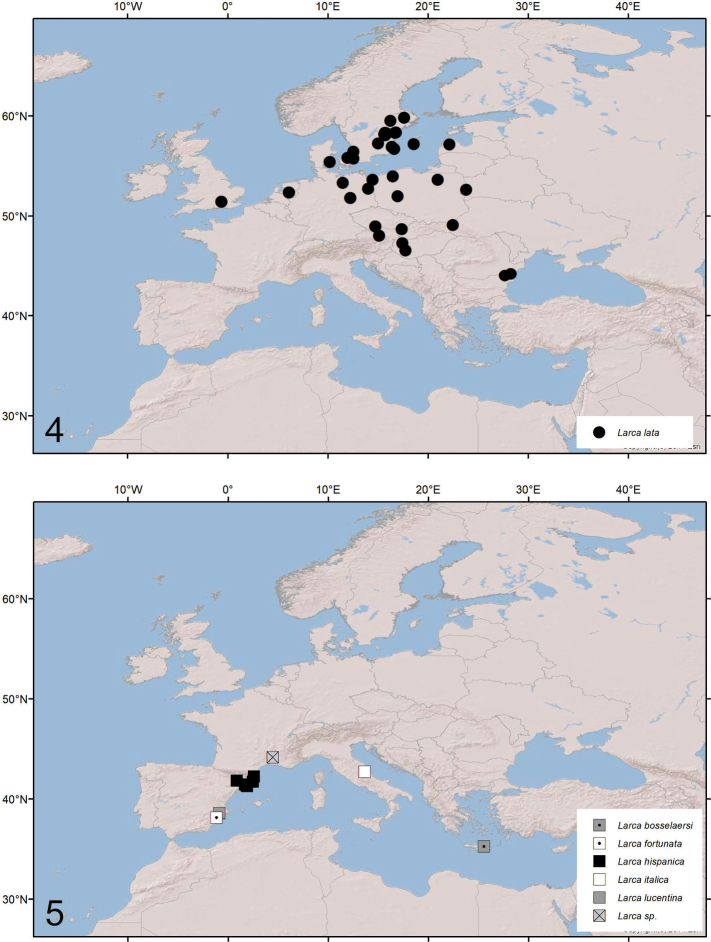
Distribution of *Larca* in Europe: **4** epigean species **5** cave-dwelling species.

A similar pattern occurs in Europe where the widespread species *L.lata* occurs sporadically throughout northern and central Europe (e.g., Lohmander 1939; Ressl and Beier 1958; Beier 1963; Dumitresco and Orghidan 1964; Ressl 1965; Beier 1970; Andersson et al. 1987; Gärdenfors and Wilander 1992; Drogla and Lippold 1994; Judson and Legg 1996; Ranius and Wilander 2000; Nilsson et al. 2001; Ranius 2001; Tooren 2001; Jansson and Hultengren 2002; Ranius 2002; Ranius and Douwes 2002; Drogla and Lippold 2004; Stol 2005; Petrov and Šťáhlavský 2007; Christophoryová et al. 2011a; Šťáhlavský 2011; Novák 2013) (Fig. 4), and the other five species are each found in one or a few caves in the Mediterranean region: *L.bosselaersi* from Milatos Cave, Crete (Henderickx and Vets 2002), *L.hispanica* in eastern Spain (Beier 1939; Estany 1980), *L.italica* from Grotta San Angelo, Italy (Gardini 1983), *L.fortunata* from Cueva del Solin, Spain (Zaragoza 2005) and *L.lucentina* from Sima del Poste, Spain (Zaragoza 2005) (Fig. 5). A cave-dwelling population of *Larca* has also been recorded from southern France (Leclerc 1979) but its identity has not been ascertained (Judson and Legg 1996).

The biogeographic patterns in North America and Europe (Figs 2–5) are extremely similar, with the epigean species usually occupying northerly habitats, and the hypogean taxa restricted to southern caves. The preponderance of cave-dwelling *Larca* species at more southerly latitudes in North America (Fig. 3) and Europe (Fig. 5) may be the result of caves becoming refuges for invertebrates where species can adapt to live in isolated subterranean habitats while the surface climate, temperature, and habitat conditions are changing over the millennia. Caves provide relatively stable temperatures and humidities compared to above-ground areas. The Pleistocene Effects Model postulates that wetter conditions during glacial periods of the Pleistocene provide connections between caves with the drier interglacial periods isolating populations and leading to genetic divergence (Barr 1968; Barr and Holsinger 1985; Derkarabetian et al. 2010). Studies of the harvestman *Sclerobunussteinmanni* Derkarabetian & Hedin, 2014 from Mallory Cave determined that *S.steinmanni* diverged from its ancestor in the late Miocene (7.2–13.4 Ma) (Derkarabetian et al. 2010). Given that the Mallory Cave harvestmen evolved to inhabit caves for at least 7 million years, it seems possible that *L.boulderica* began adapting to the cave environment in the order of a million years ago or more.

As noted by Judson and Legg (1996), species of *Larca* are xerophilic and prefer dry, dusty habitats, including tree hollows, dry caves, mammal and bird nests (Ranius et al. 2010; Turienzo et al. 2010; Christophoryová et al. 2011b; Ranius et al. 2011; Machač et al. 2018). The packrat nest remnants and the rocks where *L.boulderica* were collected were dry and dusty habitats with no moisture. The nests were on the cave floor and on a small ledge approximately 1.5 m above the floor level. There is moisture from drips and seeps further back in Mallory Cave from where the *Larca* specimens were collected. No pseudoscorpions were found in the moist parts of the cave.

### ﻿Morphological variation

Detailed examination of the eight specimens of *L.boulderica* has revealed significant intra-population variations in characters that are often cited as of inter-specific value in larcid taxonomy. Cheliceral seta *sbs* was found to be absent in most specimens leading to the presence of only four setae on the cheliceral hand. However, in two specimens, a male and a female, a fifth seta is present on one of the chelicerae. All of the species previously attributed to *Archeolarca* (*L.aalbui*, *L.cavicola*, *L.guadalupensis*, *L.rotunda* and *L.welbourni* from North America) as well as *L.chamberlini* and *L.laceyi* from North America and *L.bosselaersi* from Crete have four setae (Hoff and Clawson 1952; Benedict and Malcolm 1978; Muchmore 1981, 1984; Henderickx and Vets 2002; Harvey and Wynne 2014), and all other species, *L.granulata* and *L.notha* from North America and *L.fortunata*, *L.hispanica*, *L.italica*, *L.lata* and *L.lucentina* from Europe, usually have the full complement of five setae (Hoff 1961; Muchmore 1981; Gardini 1983; Zaragoza 2005). Zaragoza (2005) reported that specimens of *L.bosselaersi* have four or five setae on the same specimen, and among a large series of *L.hispanica* most specimens have five setae on both chelicerae; several have five setae on one chelicera and 6 on the other, and two adults had 6 setae on both chelicerae, leading him to caution against relying on cheliceral setal number to characterise species of *Larca*. The variation noted in the specimens of *L.boulderica* lends further support to that advice.

Another variable feature is the number of carapaceal setae. The holotype male of *L.boulderica* had 25 setae (arranged 6: 8: 7: 4) whereas the other male had 41 setae (10: 17: 8: 6). The four measured females had the following arrangements 6: 10: 7: 3 (= 26), 5: 13: 7: 3 (= 28), 4: 15: 7: 4 (= 30) and 6: 10: 7: 4 (= 24). Zaragoza (2005) reported similar variation in specimens of *L.lucentina* with most having four setae on the posterior margin of the carapace, but others having three, two or even one seta. Once again, caution must be taken when using this feature to characterise species of *Larca*.

## ﻿Taxonomy

**Family Larcidae Harvey, 1992**

### ﻿Genus Larca

Taxon classificationAnimaliaPseudoscorpionesLarcidae

Chamberlin, 1930

6725ABD3-3052-52AA-95D3-1467DD640530

Larca Chamberlin, 1930: 616.
Archeolarca Hoff & Clawson, 1952: 2–3.

#### Type species.

*Larca*: *Garypuslatus* Hansen, 1884, by original designation. *Archeolarca*: *Archeolarcarotunda* Hoff & Clawson, 1952, by original designation.

### ﻿Key to *Larca* species of North America

**Table:** 

1	Movable chelal finger with 4 trichobothria (Figs 14, 15)	**2**
–	Movable chelal finger with 2 or 3 trichobothria	**7**
2	Trichobothrium *ist* situated midway between *ib* and *isb*	**3**
–	Trichobothrium *ist* situated much closer to *ib* than to *isb* (Figs 14, 15)	**4**
3	Chelal hand rounded in outline (dorsal view); trichobothrium *st* separated by less than one areolar diameter from *t*	***L.cavicola* (Muchmore, 1981)**
–	Chelal hand less rounded in outline (dorsal view); trichobothrium *st* separated by at least one areolar diameter from *t*	***L.guadalupensis* (Muchmore, 1981)**
4	Chelal hand rounded in outline (dorsal view)	**5**
–	Chelal hand less rounded in outline (dorsal view) (Figs 12, 13)	**6**
5	Pedipalps larger, e.g., femur 0.90–0.995 (♂), 1.20–1.31 (♀) mm in length	***L.welbourni* (Muchmore, 1981)**
–	Pedipalps smaller, e.g., femur 0.795–0.83 (♂), 0.86–0.91 (♀) mm in length	***L.rotunda* (Hoff & Clawson, 1952)**
6	Trichobothrium *st* situated less than one areolar diameter from *t*; pedipalpal segments slender, e.g., femur 5.3–5.9 × longer than broad, patella 3.9–4.35 × longer than broad	***L.aalbui* (Muchmore, 1984)**
–	Trichobothrium *st* situated more than one areolar diameter from *t* (Figs 14, 15); pedipalpal segments less slender, e.g., femur 4.57–4.59 (♂), 4.07–4.71 (♀) × longer than broad, patella 3.09–3.22 (♂), 3.21–3.32 (♀) × longer than broad (Figs 12, 13)	***L.boulderica* sp. nov.**
7	Movable chelal finger with 2 trichobothria; larger species (e.g., pedipalpal femur greater than 0.60 mm in length)	**8**
–	Movable chelal finger with 3 trichobothria; smaller species (e.g., pedipalpal femur less than 0.55 mm in length)	***L.notha* Hoff, 1961**
8	Cheliceral hand with 5 setae, *sbs* present	***L.granulata* (Banks, 1891)**
–	Cheliceral hand with 4 setae, *sbs* absent	**9**
9	Anterior margin of carapace with 6 setae; larger species (e.g., pedipalpal femur greater than 0.85 mm in length)	***L.laceyi* Muchmore, 1981**
–	Anterior margin of carapace with 8 setae; smaller species (e.g., pedipalpal femur less than 0.80 mm in length)	***L.chamberlini* Benedict & Malcolm, 1978**

### ﻿ Larca boulderica sp. nov.

Taxon classificationAnimaliaPseudoscorpionesLarcidae

4E2E83EE-476D-5E83-BC4B-DD5AC3E7DEDA

https://zoobank.org/771E4C1C-56BD-4977-99BD-6DE45C97160A

Figs 6 , 7–9 , 10–18 , 19–21

#### Material examined.

**Types**: U.S.A.: **Colorado**: Boulder County: ***holotype*** male, Mallory Cave, 39°58.45'N, 105°17.37'W, 7000 ft (2140 m) a.s.l., 29 November 2008, under rock, dark zone of cave, D. Steinmann (DMNS). ***Paratypes***: 4 females, collected with holotype (DMNS); 1 male, collected with holotype (WAM T162363); 1 female, same data as holotype except 12 November 2009 (DMNS); 1 female, same data as holotype except 12 November 2009 (WAM T162059).

#### Diagnosis.

*Larcaboulderica* most closely resembles *L.aalbui*, *L.rotunda* and *L.welbourni* as all have four trichobothria on the movable chelal finger (Figs 14, 15) and trichobothrium *ist* is closer to *ib* than to *isb* (Figs 14, 15). The only other species with four trichobothria, *L.cavicola* and *L.guadalupensis*, have trichobothrium *ist* situated midway between *ib* and *isb. Larcarotunda* and *L.welbourni* have a rounded chelal hand, whereas *L.aalbui* and *L.boulderica* have a thinner hand (Figs 12, 13). *Larcaboulderica* differs from *L.aalbui* by the positions of trichobothria *st* and *t* (separated by at least one areolar diameter in *L.boulderica* but by less than one areolar diameter in *L.aalbui*), and the less slender pedipalpal segments [e.g., 4.57–4.59 (♂), 4.07–4.71 (♀) × and patella 3.09–3.22 (♂), 3.21–3.32 (♀) × longer than broad in *L.boulderica* (Figs 12, 13); femur 5.3–5.9 × and patella 3.9–4.35 × longer than broad in *L.aalbui*].

#### Description

**Figure 6.**
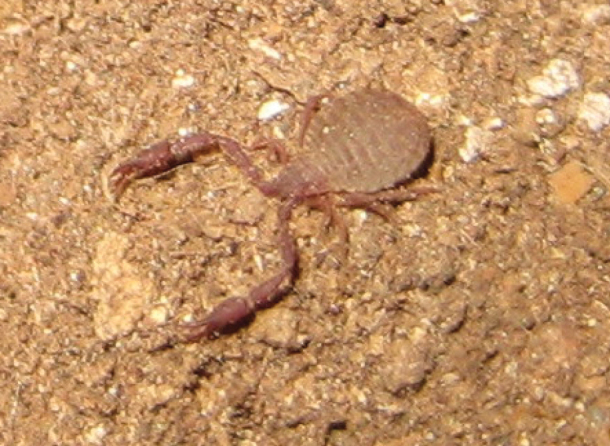
*Larcaboulderica* sp. nov., in situ in Mallory Cave.

**Figures 7–9.**
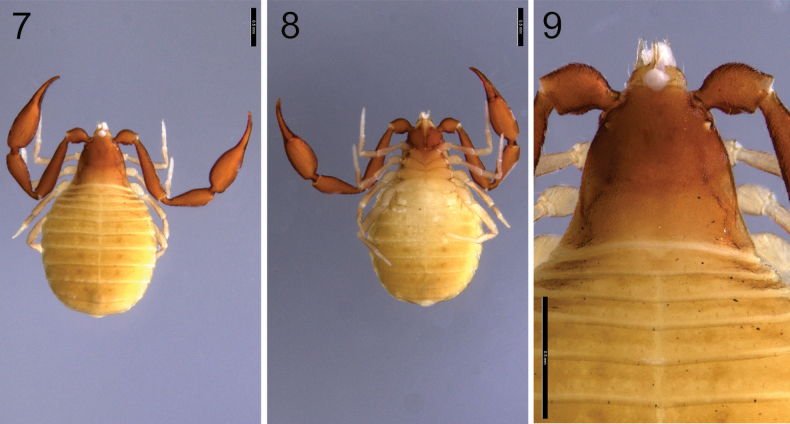
*Larcaboulderica* sp. nov., paratype female (DMNS): **7** body, dorsal **8** body, ventral **9** cephalothorax, dorsal. Scale bars: 0.5 mm.

**(adults). *Colour***: most body parts pale yellow-brown, genital region of female and legs slightly paler (Figs 6–9).

***Setae and cuticle***: setae long, usually curved, distally acuminate; most cuticular surfaces granulate.

***Chelicera***: with 4 (rarely 5) setae on hand, with *sbs* usually absent, and 1 subdistal seta on movable finger; all setae acuminate; seta *bs* slightly shorter than others; with 2 dorsal lyrifissures and 1 ventral lyrifissure; galea of ♂ short with terminal bifurcation (Fig. 16), of ♀ long and slender with 3 terminal to subterminal rami (Fig. 17); rallum of 4 blades, the most distal blade with several serrations on leading edge, other blades smooth; serrula exterior with 16 (♂), 18 (♀) blades; lamina exterior present.

***Pedipalps***: Pedipalp (Figs 12, 13): most surfaces of trochanter, femur, patella and chelal hand lightly and granulate, chelal fingers smooth; trochanter, femur, patella and chelal hand with prominent, curved, slightly denticulate setae arranged sparsely; patella with 3 small sub-basal lyrifissures; trochanter 1.85 (♂), 1.79 (♀), femur 4.57–4.59 (♂), 4.07–4.71 (♀), patella 3.09–3.22 (♂), 3.21–3.32 (♀), chela (with pedicel) 4.36–5.00 (♂), 3.83–4.03 (♀), chela (without pedicel) 4.09–4.78 (♂), 3.60–3.81 (♀), hand (with pedicel) 2.09–2.33 (♂), 1.83–1.87 (♀) × longer than broad, movable finger (with pedicel) 0.96–1.05 (♂), 0.97–1.03 (♀) × longer than hand. Fixed chelal finger with 8 trichobothria, movable chelal finger with 4 trichobothria (Figs 14, 15): *eb*, *esb*, *ib* and *ist* situated subbasally, *est*, *isb* and *it* submedially, *et* subdistally, *est* slightly distal to *it*, *ib* opposite *esb*, and *ist* distal to *esb*; *b* and *sb* situated subbasally, and *st* and *t* situated submedially, with *st* situated very close to *t*, separated by slightly more than 1 areolar diameter; patch of microsetae not present on retrolateral margin of fixed chelal finger near et. Venom apparatus present in both chelal fingers, venom ducts not visible. Chelal teeth rounded, very low; fixed finger with 30 (♂), 30 (♀) teeth; movable finger with 29 (♂), 28 (♀) teeth; accessory teeth absent.

**Figures 10–18.**
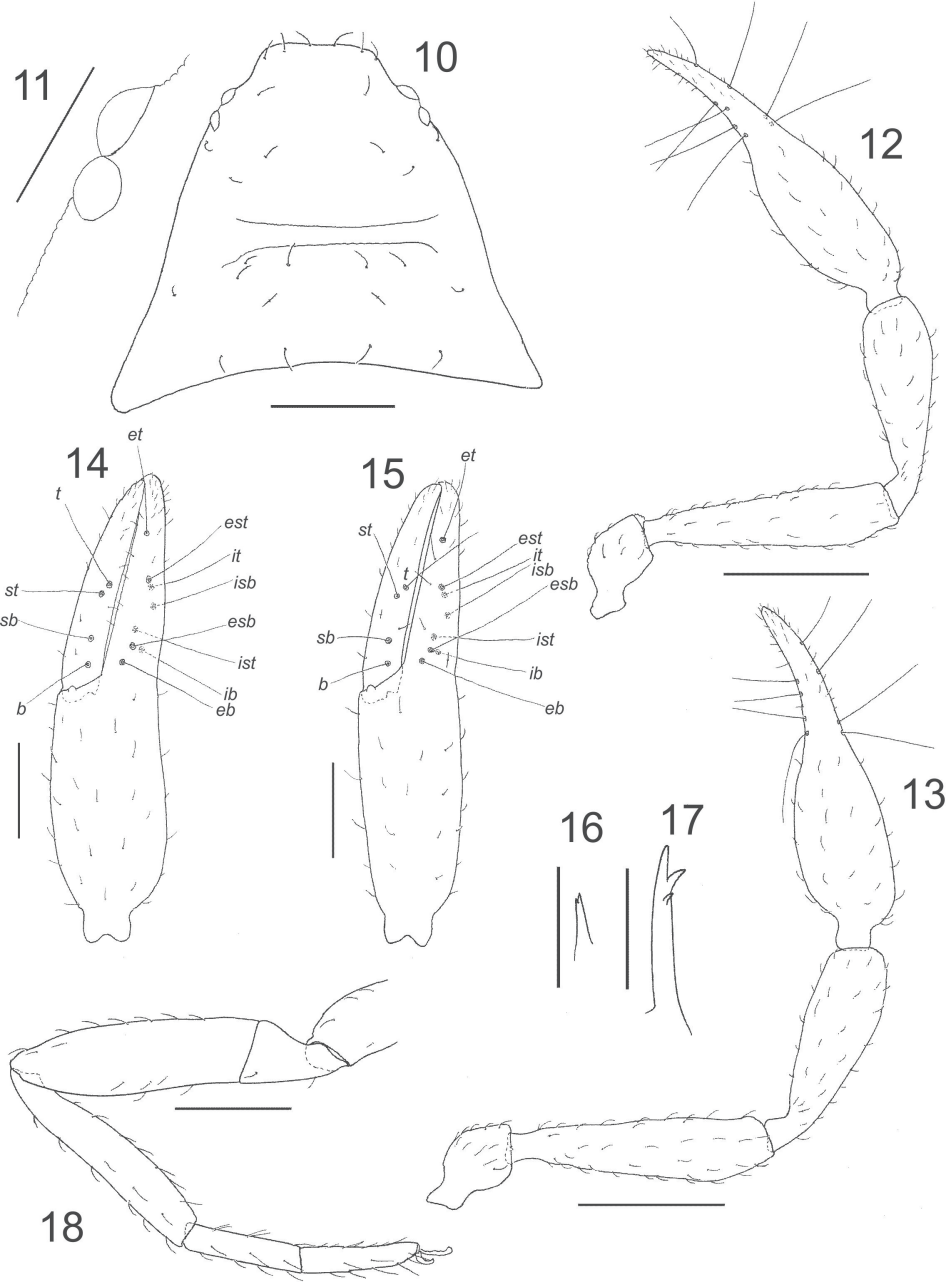
*Larcaboulderica* sp. nov., holotype male and paratype female (DMNS): **10** carapace, dorsal, male **11** left pair of eyes, dorsal, male **12** right pedipalp, dorsal, male **13** right pedipalp, dorsal, male **14** left chela, retrolateral, male **15** left chela, retrolateral, female **16** left galea, dorsal, male **17** left galea, ventral, female **18** left leg IV, retrolateral, male. Scale bars: 0.5 mm (**12, 13**); 0.25 mm (**10, 14, 15, 18**); 0.1 mm (**11**); 0.05 mm (**16, 17**).

***Cephalothorax***: carapace (Figs 9, 10): 0.73–0.75 (♂), 0.79–0.83 (♀) × longer than broad; anterior margin straight; with 2 pairs of rounded corneate eyes, tapetum present; with 25–41 (♂), 24–30 (♀) setae, arranged with 6–8 (♂), 4–6 (♀) near anterior margin, 8–17 (♂), 10–15 (♀) in prozone, 7–8 (♂), 7 (♀) in metazone and 4–6 (♂), 3–4 (♀) near posterior margin; with 1 deep, broad median furrow. Coxal region: manducatory process rounded with 1 distal seta, 1 small sub-oral seta, and 12 (♂), 9 (♀) additional setae; median maxillary lyrifissure large, rounded and situated submedially; posterior maxillary lyrifissure rounded. Coxae I to IV becoming progressively wider. Chaetotaxy of coxae I–IV: ♂, 8: 8: 8: 12; ♀, 8: 8: 7: 11.

***Legs***: femora I and II longer than patellae; junction between femora and patellae III and IV very angulate; femora III and IV much smaller than patellae III and IV; femur + patella of leg IV 5.21 (♂), 4.81 (♀) × longer than broad (Fig. 18); metatarsi and tarsi not fused; tarsus IV without tactile seta; subterminal tarsal setae arcuate and acuminate; claws simple; arolium much longer than claws, not divided.

**Figures 19–21.**
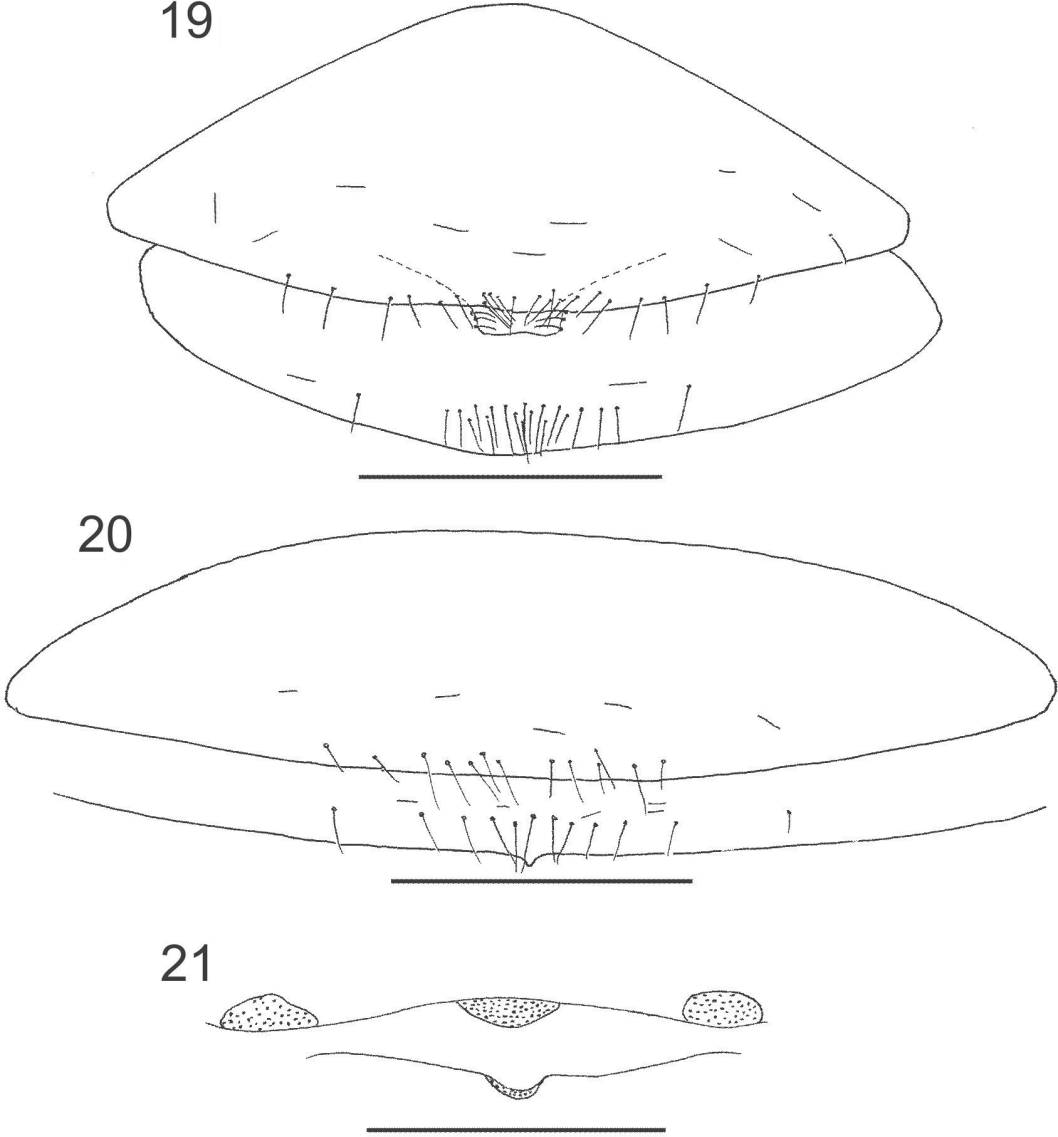
*Larcaboulderica* sp. nov., holotype male and paratype female (DMNS): **19** genital sternites, ventral, male **20** genital sternites, ventral, female **21** genitalia, ventral, female. Scale bars: 0.2 mm.

***Abdomen***: tergites II–VIII and sternites IV–VIII of male and female with medial suture line fully dividing each sclerite. Tergal chaetotaxy: ♂, 7: 8: 8: 11: 11: 11: 11: 10: 6: 6 (arranged T4T): 6: 2; ♀, 4: 6: 10: 11: 12: 11: 12: 11: 10: 8 (arranged T6T): 4: 2; tergites I–X uniseriate. Sternal chaetotaxy: ♂, 22: (0) 7 [3 + 3] (0): (0) 21 (0): 8: 9: 8: 8: 6: 6: 4: 2; ♀, 13: (0) 12 (0): (0) 7 (0): 8: 7: 8: 9: 7: 6: 4: 2; sternites IV–X uniseriate; ♂ and ♀ sternite II with all setae situated near posterior margin (Figs 19, 20); most setae of male sternite III clustered near posterior margin (Fig. 19). Spiracles with helix. Anal plates (tergite XII and sternite XII) situated between tergite XI and sternite XI, and surrounded by desclerotized region of tergite XI and sternite XI; sternite XI with 26 (♂), 22 (♀) small lyrifissures. Pleural membrane finely wrinkled-plicate; without any setae.

***Genitalia***: male: very similar to that described for *L.laceyi* Muchmore, 1981 by Muchmore (1981). Female with 1 pair of lateral cribriform plates and 2 median cribriform plates, one of which is larger than the other (Fig. 21); spermathecae absent.

***Dimensions* (mm)**: Male: holotype, with 1 other male in parentheses (when measured): Body length (excluding chelicerae) 2.37 (2.25). Pedipalp: trochanter 0.370/0.200, femur 0.895/0.195 (0.845/0.185), patella 0.725/0.225 (0.650/0.210), chela (with pedicel) 1.200/0.275 (1.225/0.245), chela (without pedicel) length 1.200 (1.170), chelal hand (without pedicel) length 0.575 (0.570), movable finger length 0.550 (0.600). Carapace 0.640/0.850 (0.580/0.800), anterior eye diameter 0.055, posterior eye diameter 0.050. Leg IV: femur + patella 0.730/0.140, tibia 0.530/0.100, metatarsus 0.265/0.065, tarsus 0.250/0.055.

Female: paratype, with 3 other females in parentheses (when measured): Body length (excluding chelicerae) 2.51 (2.51–2.74). Pedipalp: trochanter 0.375/0.210, femur 0.895/0.205 (0.895–0.990/0.205–0.220), patella 0.755/0.235 (0.765–0.795/0.230–0.240), chela (with pedicel) 1.205/0.315 (1.190–1.255/0.295–0.325), chela (without pedicel) length 1.135 (1.125–1.255), chelal hand (without pedicel) length 0.575 (0.550–0.595), movable finger length 0.565 (0.565–0.575). Carapace 0.640/0.815 (0.645–0.655/0.805–0.815), anterior eye diameter 0.065, posterior eye diameter 0.060. Leg IV: femur + patella 0.745/0.155, tibia 0.530/0.095, metatarsus 0.270/0.065, tarsus 0.250/0.055.

#### Etymology.

The species epithet is a noun taken from the type locality of Boulder County, Colorado. Mallory Cave is situated on City of Boulder, Open Space and Mountain Parks property.

## Supplementary Material

XML Treatment for Larca

XML Treatment for Larca boulderica

## ﻿Additional information

### Author ORCIDs

Mark S. Harvey https://orcid.org/0000-0003-1482-0109

### Data availability

All of the data that support the findings of this study are available in the main text.
